# Annotations

**Published:** 1908-10-24

**Authors:** 


### Annotations.

### The New British Pharmacopœia.

Dr. Nestor Tirard, in his recent address before the Therapeutical and Pharmacological Section of the Royal Society of Medicine upon "The British Pharmacopœia: Its Scope and Objects," gave some interesting details of this important work, and incidentally referred to the steps which are now being taken in view of the forthcoming revised edition. He outlined the factors which from time to time have served to widen the scope of the British Pharmacopœia from that which was originally laid down in the Medical Act of 1858; and he pointed out the need for further changes in the future, whereby greater uniformity may be secured between the pharmacopœias of the various nations. With regard to the new issue, Dr. Tirard stated that great progress has already been made with many points, involving prolonged researches. These were undertaken very shortly after the publication of 1898. The Pharmaceutical Societies of Great Britain and of Ireland were invited to appoint representatives to confer with members of the Pharmacopœia Committee of the General Medical Council, and numerous subjects requiring investigation have been submitted to various experts in pharmacy and pharmaceutical chemistry. Dr. Tirard referred to the steps taken to ascertain what omissions, additions, and alterations in the Pharmacopœia are desired by medical men and by pharmacists, and to the reports received from the different medical authorities, from the Therapeutic Committee of the British Medical Association, from a collation of recent prescriptions, and from a committee of wholesale druggists. The details of most of these are at present confidential. The difficulties likely to be entailed by the introduction of sera, or of the physiological standardisation of drugs were also referred to, since these would seem to involve the creation of special State laboratories, and the substitution of an official guarantee in place of personal responsibility. As might be expected, Dr. Tirard announced that, in view of the number and diversity of the suggestions received, it would be impossible to act upon them all, but that he hoped the new Pharmacopœia would show that much has been done by good intentions and by mutual concessions.

### The Tuberculosis Congress.

From accounts at present available, the proceedings neither at Washington nor at Philadelphia seem to have been very eventful. Apparently Professor Koch still thinks that bovine tuberculosis is not responsible for any considerable amount of human infection. He seems, however, to have been hard put to it to defend his views at the point of attack chosen by his opponents. Dr. Newsholme spoke at some length on his contention that institutional segregation of consumptives is the only means that can be regarded as bearing a causal relation to the admitted decline in tuberculosis. During the proceedings two complaints of rather unequal importance have been made. The arrangements for the delegates, contrary to the traditions of American hospitality, were perhaps a little neglected. Further, it is hinted that responsible lay influences prevented American physicians from coming into line with their *confrères* as regards certain resolutions directed against unhygienic buildings. British successes at the exhibition held in connection with the Congress comprise a gold medal to the English pathological exhibit, two prizes to the Brompton Hospital—for exhibits dealing respectively with the treatment of advanced consumptives and of curable cases among the working classes—and an award to the Woman's Health Association of Ireland, for the best work done by a voluntary association against tuberculosis since the last Congress. This prize was shared with the Charity Organisation of New York. Germany won the gold medal, open to foreign countries, for exhibits illustrating the restriction of tuberculosis.

### Criminal Abortion in America.

There is some very plain speaking on the subject of criminal abortion in the *American Journal of Obstetrics* from the pen of Dr. F. H. Jackson. Putting it at a conservative estimate, he believes there are 50,000 such crimes committed annually within the State of Maine alone, and he does not hesitate to add that the majority of these are carried out, not by midwives or unqualified practitioners, but by members of the medical profession. In a vigorous denunciation of this state of things, which is a revelation indeed, and one for which no language is too strong, he says that several of these gentry are members of the Maine Medical Association, and are prominent socially and in church work. This estimate of the alarming prevalence of criminal abortion in the United States is borne out by Dr. Dorsett's recent address before the obstetrical section of the American Medical Association. Although he puts down a portion of the evil to the fixed determination of many women to avoid maternity at any cost, Dr. Jackson is earnest that, for the honour and dignity of the medical profession, concerted action is required to drive the practitioners who aid and abet the offence from the country. It appears that to procure abortion, and to assist or attempt such an act, is not a felony in American law—a curious laxity in the legal code, which is probably as much responsible as anything else for the disclosed facts. It further appears that some judge has lately sentenced a man proved guilty of two abortions to three years', and a tramp found guilty of stealing a small sum of money, to five years' imprisonment respectively. The author deals fully with the treatment of sepsis and the other possible complications of criminal abortion, but perhaps the most important part of his advice, and a point upon which he lays great stress, is the paramount necessity of never attending such a case except in consultation with another practitioner of repute. Dr. Jackson knows of more than one case, he says, in which the real abortionist has attempted to foist the blame of his deeds on to the shoulders of the physician called in to repair the consequences of his *mala praxis*. This is indeed a point worth bearing in mind by every general practitioner and obstetrician.

